# Supplementary material for: microRNA Biomarkers in Paediatric Infection Diagnostics—Bridging the Gap Between Evidence and Clinical Application: A Scoping Review
Source: Noncoding RNA. 2025 Sep 24;11(5):71. doi: 10.3390/ncrna11050071 (PMC12566583; doi:10.3390/ncrna11050071)
Supplement: Supplementary file 1 [file ncrna-11-00071-s001.zip › ncrna-3816475-supplementary.pdf]

Search Strategy

The search strategy was adapted for each database.

Medline All search:

miRNA.mp. MicroRNAs/ AND infection.mp. OR infections/ OR infectious disease.mp. OR bacterial infection.mp OR bacterial infections/ OR viral infection.mp OR virus diseases/ AND children.mp. OR child/ OR Infant/ or Adolescent/ or Pediatrics/ or paediatrics.mp. or Child, Preschool/ OR Neonatal Sepsis/ OR Intensive Care, Neonatal/ OR Intensive Care Units, Neonatal/ OR neonatal.mp.

Web of Science Core Collection search:

microrna* or miRNA* (All Fields) and infecti* or bacteri* or virus* or viral (All Fields) and child* or infant* or adolescent or p$ediatric* (All Fields) and Article (Document Types) and English (Languages)
